# Baseline NT‐proBNP Accurately Predicts Symptom Response to Transcatheter Aortic Valve Implantation

**DOI:** 10.1161/JAHA.120.017574

**Published:** 2020-11-26

**Authors:** Christopher J. Allen, Jubin Joseph, Tiffany Patterson, Matthew Hammond‐Haley, Hannah Z. R. McConkey, Bernard D. Prendergast, Michael Marber, Simon R. Redwood

**Affiliations:** ^1^ Cardiovascular Division St. Thomas Hospital King's College London London United Kingdom; ^2^ Department of Cardiology Guys’ and St Thomas NHS Foundation Trust London United Kingdom

**Keywords:** aortic stenosis, NT‐proBNP, transcutaneous aortic valve implantation, transfemoral aortic valve implantation, Aortic Valve Replacement/Transcather Aortic Valve Implantation, Quality and Outcomes, Valvular Heart Disease

## Abstract

**Background:**

Up to 30% of patients undergoing transcatheter aortic valve implantation (TAVI) experience minimal symptomatic benefit or die within 1 year, indicating an urgent need for enhanced patient selection. Previous analyses of baseline NT‐proBNP (N‐terminal pro‐brain natriuretic peptide) and TAVI outcomes have assumed a linear relationship, yielding conflicting results. We reexamined the relationship between baseline NT‐proBNP and symptomatic improvement after TAVI.

**Methods and Results:**

Symptom status, clinical and echocardiographic data, and baseline NT‐proBNP were reviewed from 144 consecutive patients undergoing TAVI for severe symptomatic aortic stenosis. The primary end point was change in New York Heart Association functional class at 1 year. There was a nonlinear, inverted‐U relationship between log‐baseline NT‐proBNP and post‐TAVI change in NYHA class (*R* ^2^=0.4559). NT‐proBNP thresholds of <800 and >10 000 ng/L accurately predicted no symptomatic improvement at 1 year (sensitivity 88%, specificity 83%, positive predictive value 72%, negative predictive value 93%). In adjusted analyses, baseline NT‐proBNP outside this “sweet‐spot” range was the only factor independently associated with poor functional outcome (high: NT‐proBNP >10 000 ng/L, odds ratio [OR], 65; 95% CI, 6–664; low: NT‐proBNP <800 ng/L, OR, 73; 95% CI, 7–738).

**Conclusions:**

Baseline NT‐proBNP is a useful prognostic marker to predict poor symptom relief after TAVI and may indicate when intervention is likely to be futile. Both low (<800 ng/L) and very high (>10 000 ng/L) levels are strongly associated with poor functional outcome, suggesting an alternative cause for symptoms in the former scenario and an irrevocably diseased left ventricle in the latter. Further evaluation of this relationship is warranted.

**Nonstandard Abbreviations and Acronyms**
AS: aortic stenosis
CCS: Canadian Cardiovascular Society
NYHA: New York Heart Association
TAVI: transcatheter aortic valve implantation

**Clinical Perspective**

**What Is New?**

- In this cohort of 148 patients with severe symptomatic aortic stenosis undergoing transcatheter aortic valve implantation, baseline NT‐proBNP (N‐terminal pro–brain natriuretic peptide) demonstrated a nonlinear, inverted‐U relationship with symptomatic relief (New York Heart Association functional class) at 1 year.
- Both low (<800 ng/L) and very high (>10 000 ng/L) baseline NT‐proBNP levels were strongly associated with poor functional outcome, retaining an independent association in adjusted analyses and predicting futility of symptom relief with a high degree of diagnostic accuracy.
- Low values suggested an alternative cause for presenting symptoms (demonstrating an association with chronic lung disease), whereas very high values correlated with irrevocable cardiac damage (left ventricular systolic dysfunction, diastolic impairment, pulmonary hypertension, and concomitant valve disease).

**What Are the Clinical Implications?**

- Baseline NT‐proBNP may be a useful, pragmatic marker to predict futility of symptom relief after transcatheter aortic valve implantation, a scenario prevalent in up to a third of patients despite excellent immediate procedural results (>95% technical success).

Transcatheter aortic valve implantation (TAVI) transformed the management of severe calcific aortic stenosis (AS) in symptomatic patients at high or prohibitive surgical risk, reducing morbidity and mortality in the face of an otherwise grave prognosis.^1^, ^2^, ^3^ The procedure is now an established standard of care for the majority of patients with severe symptomatic AS.^4^, ^5^, ^6^, ^7^ Despite clear benefits in defined populations, there remains marked heterogeneity in outcome at the individual patient level, with up to 30% experiencing minimal symptomatic benefit or dying within 1 year of intervention, regardless of immediate outcome.^8^ Enhanced patient selection and risk stratification are therefore pressing requirements to enable targeted assessment of individual risk, informed shared decision making, and the avoidance of unnecessary, expensive, and potentially harmful (“futile”) procedures in those unlikely to benefit.^9^

Secreted by the myocardium in response to increased mechanical wall stress, blood levels of BNP (brain natriuretic peptide) and its pro‐hormone NT‐proBNP (N‐terminal proBNP) correlate positively with severity of AS and symptom onset, and elevated levels tend to reflect advanced disease.^10^, ^11^, ^12^ Accordingly, international practice guidelines advocate their role in patient selection, particularly in those with severe AS and equivocal symptoms.^13^

Prior studies have examined the association between baseline natriuretic peptide levels and mortality after TAVI, although results have been inconsistent with discordant findings.^14^, ^15^, ^16^, ^17^, ^18^, ^19^, ^20^ In contrast, there is a surprising paucity of data examining the relationship between baseline BNP/NT‐proBNP and subsequent change in symptomatic or functional status.^15^, ^18^ In both scenarios, a linear relationship with outcome has been assumed. This retrospective observational study reexamined the relationship between baseline NT‐proBNP level and symptomatic improvement 1 year after TAVI.

## Methods

The data that support the findings of this study are available from the corresponding author upon reasonable request. Data collection within the National Health Service is performed without explicit consent for provision of healthcare, administrative, and clinical audit purposes (local or national) and is performed under the auspices of European Law—General Data Protection Regulation (GDPR) Act (May 2018): article 6(1)(e); 9(2)(h); special category 9(2)(i). These data were extracted retrospectively for assessment of healthcare quality, delivery, and outcomes, and therefore separate institutional review board approval was not required for this work.

### Study Design and Patient Population

Symptom status (New York Heart Association [NYHA] class, Canadian Cardiovascular Society class, syncope) at baseline and 1‐year follow‐up, clinical characteristics, baseline NT‐proBNP level, and echocardiographic indices were retrospectively reviewed from a prospectively maintained database of consecutive patients undergoing TAVI at a high volume UK institution over a 1‐year period (January 1, 2015 to January 1, 2016). All patients had symptomatic severe native AS (aortic valve area ≤0.8 cm^2^ or mean gradient >40 mm Hg)^21^ and were deemed at prohibitive or high risk for conventional surgical aortic valve replacement by a multidisciplinary heart team.

The primary end point was change in NYHA functional class (∆NYHA) at 1 year, calculated by subtracting 1 year NYHA from baseline NYHA. Patients who died within a month of TAVI (n=4, 3%) were excluded from analysis. Those who survived >30 days but died within 1 year were coded as ∆NYHA=0 (no improvement, n=17, 11%).

### NT‐proBNP Sampling

Baseline NT‐proBNP samples were obtained before TAVI from an antecubital or other accessible peripheral vein into EDTA‐containing tubes and processed in standard fashion. NT‐proBNP level was measured using a chemoluminescent immunoassay (Elecsys proBNP II; Roche, Minneapolis, Minnesota, United States).

### Echocardiography

Baseline transthoracic echocardiography was performed 3 months before TAVI and at 1‐year follow‐up using standard, commercially available consoles and analyzed in accordance with contemporary practice recommendations.^22^, ^23^, ^24^, ^25^, ^26^ Peak aortic jet velocity was estimated via continuous wave Doppler from the apical 3‐ or 5‐chamber views, with mean and peak transvalvular pressure gradients derived using the Bernoulli equation.^22^ Aortic valve area was computed from the velocity time integrals of the left ventricular (LV) outflow tract and aortic valve using the continuity equation.^22^ LV dimensions were assessed in the parasternal long‐axis view, with LV mass calculated using the Devereux formula and indexed to body surface area (left ventricular mass index)^23^ and LV ejection fraction calculated using Simpson's biplane method after measurement of LV end‐diastolic and end‐systolic volumes in the apical 2‐ and 4‐chamber views.^23^ Mitral, aortic, and tricuspid valve regurgitation was evaluated using spectral and color Doppler images and graded using a multiparametric approach.^24^ Where feasible, LV diastolic function and estimated LV filling pressures were characterized and graded in accordance with contemporary recommendations.^25^ Systolic pulmonary artery pressure was estimated as the sum of the tricuspid valve pressure gradient and estimated right atrial pressure (determined by inferior vena cava assessment).^26^ Longitudinal right ventricular function was assessed through tricuspid annular plane systolic excursion measured via anatomical M‐mode in a focused apical 4‐chamber view of the right ventricle.^26^

### Statistical Analysis

Categorical variables are presented as frequencies and percentages. Continuous data are presented as mean±SD or median (interquartile range), as appropriate. After distribution assessment, differences between continuous variables were tested for significance by analysis of variance or Kruskal–Wallis test for normally and non‐normally distributed data, respectively. Categorical variables were compared using the chi‐square test. For multiple comparisons, Bonferroni corrections were employed in post hoc analyses. The prognostic discrimination of baseline NT‐proBNP level was assessed by generating receiver operating characteristics curves and chi‐square test used to determine the equality of the receiver operating characteristics‐estimated area under the curve. Multivariable linear regression analysis was performed to determine the association of the defined NT‐proBNP cut‐points and other baseline clinical and echocardiographic parameters with the primary outcome. Included in the multivariable model were the following: estimated glomerular filtration rate, left ventricular ejection fraction, estimated left ventricular filling pressure, pulmonary artery systolic pressure, chronic lung disease, atrial fibrillation, ≥ moderate mitral regurgitation, ≥ moderate tricuspid regurgitation, and right ventricular dysfunction. After initial univariable regression modeling, any additional variables exhibiting a *P*<0.05 were also included in the multivariable model. Odds ratios (ORs) with 95% CIs are presented. Two‐sided *P*<0.05 were considered statistically significant. All analyses were performed using SPSS software version 23.0 (IBM, Armonk, New York).

## Results

### Baseline Characteristics

A total of 148 patients underwent TAVI over the inclusion period, of whom 4 (3%) died within 30 days of TAVI and were excluded from analysis. Baseline clinical, echocardiographic, and procedural data for the 144 patients included in the analysis are summarized in Table 1. Mean baseline NT‐proBNP was 5305±8886 ng/L. The majority of patients underwent transfemoral TAVI with balloon expandable prostheses. Mean NYHA functional class was 2.6±0.7 and 1.8±0.9 at baseline and 1 year post‐procedure, respectively. One‐year mortality was 11% (17 patients). Forty‐six patients (32%) experienced no improvement in NYHA functional class (∆NYHA ≤0) at 1 year or died between 30 days and 1 year after TAVI and were defined as treatment nonresponders.

**Table 1:** Baseline Characteristics

	n=144
Demographics	
Age, y	83±7
Female	72 (50%)
Body mass index, kg/m^2^	28±6
Past medical history	
Hypertension	120 (83%)
Diabetes mellitus	34 (24%)
Coronary artery disease	68 (47%)
Previous myocardial infarction	27 (19%)
Previous percutaneous coronary intervention	23 (16%)
Previous coronary artery bypass graft	37 (26%)
Peripheral arterial disease	29 (20%)
Cerebrovascular disease	15 (10%)
Chronic lung disease	35 (24%)
Atrial fibrillation	34 (24%)
Presenting symptoms	
New York Heart Association III–IV	94 (65%)
Syncope	19 (13%)
Canadian Cardiovascular Society III–IV	7 (5%)
Echocardiography	
Left ventricular ejection fraction, %	54±11
Aortic valve area, cm^2^	0.7±0.2
Mean gradient, mm Hg	49±16
Maximal gradient, mm Hg	72±21
Laboratory values	
Estimated glomerular filtration rate, mL/min	59±23
N‐terminal pro‐brain natriuretic peptide, ng/L	5305±8886
Procedural characteristics	
Transfemoral	118 (82%)
Balloon expandable valve	127 (88%)
Self‐expanding valve	17 (12%)

### Relationship Between Baseline NT‐proBNP and ∆NYHA Functional Class 1 Year After TAVI

**Figure 1:**
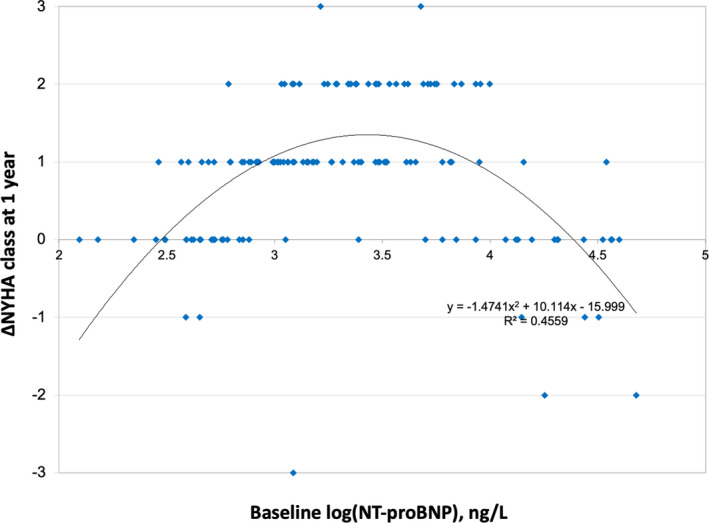
Association between log‐transformed baseline NT‐proBNP change in NYHA functional class at 1 year after transcatheter aortic valve implantation. NT‐proBNP indicates N‐terminal pro‐brain natriuretic peptide; and NYHA, New York Heart Association.

Baseline NT‐proBNP values were positively skewed (mean±SD=5305±8886 ng/L, median±interquartile range=1800±4296 ng/L) and normalized via logarithmic transformation. There was a nonlinear, inverted‐U relationship between log‐transformed baseline NT‐proBNP and change in NYHA functional class 1 year following TAVI (Figure 1). Multiple polynomial regression lines were modeled, with a quadratic curve (Figure 1, *R* ^2^=0.4559, *x*‐axis intercepts 298 and 24 396 ng/L) ultimately demonstrating the best fit, superior to cubic (*R* ^2^=0.4116), quartic (*R* ^2^=0.4032), quintic (*R* ^2^=0.4076), and other higher order polynomials.

### Identification of NT‐proBNP Cutoff Values Associated With Symptomatic Improvement 1 Year After TAVI

**Figure 2:**
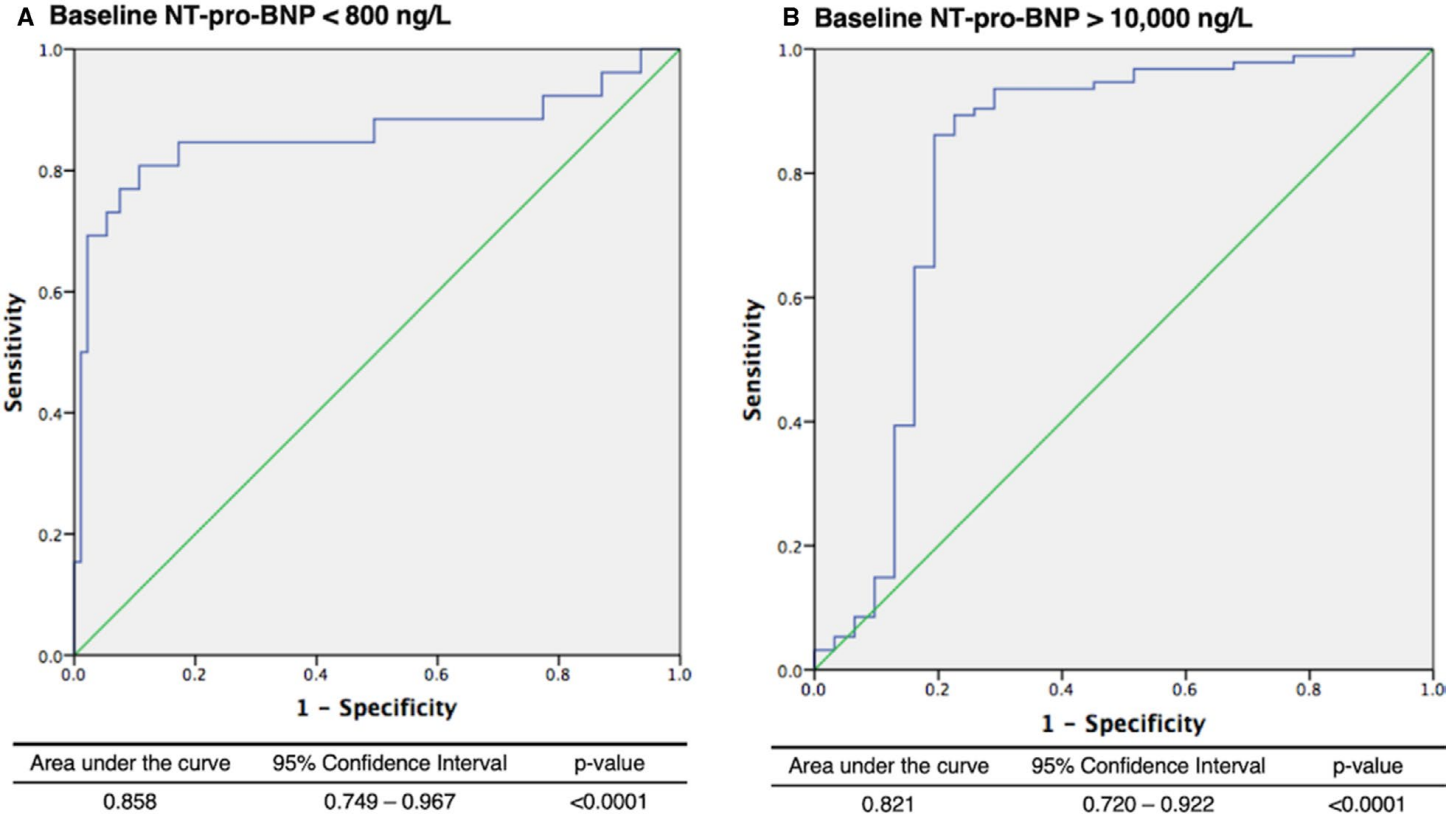
Receiver operating characteristic curves demonstrating the diagnostic performance of (**A**) baseline NT‐proBNP <800 ng/L and (**B**) baseline NT‐proBNP >10 000 ng/L to highlight treatment nonresponse to TAVI at 1‐year follow‐up, defined as no improvement in New York Heart Association functional class. NT‐proBNP indicates N‐terminal pro‐brain natriuretic peptide; and TAVI, transcatheter aortic valve implantation.

Received operator characteristic curve analysis was performed to identify clinically relevant cutoff values. A baseline NT‐proBNP of <800 and >10 000 ng/L accurately predicted no improvement in NYHA functional class at 1 year (Figure 2A and B), with an area under the curve of 0.858 (95% CI, 0.749–0.967; *P*<0.0001) and 0.821 (95% CI, 0.720–0.922; *P*<0.0001), respectively. The precision of the intermediate baseline NT‐proBNP range (800 ng/L < NT‐proBNP <10 000 ng/L) to predict no improvement was accordingly much lower (area under the curve 0.267; 95% CI, 0.048–0.485). Baseline NT‐proBNP outside this range (<800 or >10 000 ng/L) predicted the absence of a response to treatment with high diagnostic accuracy (sensitivity 88%, specificity 83%, positive predictive value 72%, negative predictive value 93%).

### Differences Between TAVI Responders and Nonresponders

Differences in baseline clinical and echocardiographic characteristics between treatment responders and nonresponders, grouped according to low (<800 ng/L); midrange (≥800 ng/L ≤10 000 ng/L); and high (>10 000 ng/L) baseline NT‐proBNP are summarized in Table 2. The majority of 98 TAVI responders (87%, n=85) fell into the midrange NT‐proBNP category, with relatively few outside the low (11%, n=11) and high (2%, n=2) NT‐proBNP thresholds (Figure S1). Chronic lung disease was more common in treatment nonresponders with low NT‐proBNP. TAVI nonresponders with high baseline NT‐proBNP had worse baseline renal function and LV systolic function, with higher LV filling pressures and increased pulmonary pressures. Concomitant valve disease was also more common in TAVI nonresponders with high baseline NT‐proBNP, with a higher prevalence of significant mitral regurgitation, tricuspid regurgitation, and right ventricular dysfunction.

**Table 2:** Between Group Baseline Differences of TAVI Symptomatic Responders Versus Nonresponders

	Responders	Nonresponders (All)	Nonresponders (Grouped by NT‐proBNP Category)			*P* Value	Adjusted *P* Value
			Low NT‐proBNP	Midrange NT‐proBNP	High NT‐proBNP		
	n=98	n=46	NT‐proBNP <800 ng/L (n=23, 50%)	800 < NT‐proBNP <10 000 ng/L (n=6, 13%)	NT‐proBNP >10 000 ng/L (n=17, 37%)		
Baseline NYHA functional class	2.7±0.6	2.4±0.7	2.0±0.7	2.5±0.6	3.0±0.5		
NYHA functional class 12 mo post‐TAVI	1.4±0.5	2.7±0.5	2.1±0.8	2.6±0.7	3.2±0.6		
Female	49 (50%)	23 (50%)	10 (43%)	4 (67%)	9 (53%)	0.773	
Age, y	83±6	82±6	80±10	82±9	85±4	0.302	
Body mass index, kg/m^2^	28±7	27±5	28±4	28±6	25±5	0.463	
**Estimated glomerular filtration rate, mL/min**	60±24	55±23	66±18	48±21	43±21	0.003*	0.024^†^
Hypertension	83 (85%)	37 (80%)	19 (83%)	5 (83%)	13 (76%)	0.978	
Diabetes mellitus	22 (22%)	12 (26%)	6 (26%)	3 (50%)	3 (18%)	0.435	
Coronary artery disease	44 (45%)	24 (52%)	12 (52%)	3 (50%)	9 (53%)	0.878	
Peripheral arterial disease	20 (20%)	9 (20%)	5 (22)	1 (17%)	3 (18%)	0.169	
Cerebrovascular disease	11 (11%)	4 (9%)	0 (0%)	1 (17%)	3 (18%)	0.758	
**Chronic lung disease**	18 (18%)	17 (37%)	12 (52%)^†^	2 (33%)	3 (18%)	0.007*	0.001^†^
Atrial fibrillation	26 (26%)	13 (28%)	5 (22%)	2 (33%)	6 (35%)	0.084	
**Left ventricular ejection fraction**	55±10	53±11	59±5	49±12	45±12	<0.0001*	0.003^†^
Aortic valve area, cm^2^	0.7±0.2	0.7±0.2	0.7±0.2	0.6±0.2	0.6±0.1	0.446	
Mean gradient, mm Hg	48±15	51±19	50±18	60±19	50±21	0.562	
Peak gradient, mm Hg	72±22	73±20	70±19	85±32	74±18	0.659	
**Estimated left ventricular filling pressure**	16±8	18±8	14±7	17±3	23±6	0.001*	0.003^†^
**≥ Grade II diastolic dysfunction**	30 (31%)	21 (45%)	6 (26%)	3 (50%)	12 (71%)	0.036*	NS
Left ventricular mass index, g/m^2^	120±35	119±39	101±23	126±49	134±44	0.082	
Left atrial enlargement	68 (69%)	31 (67%)	9 (39%)	6 (100%)	16 (94%)	0.099	
**≥ moderate mitral regurgitation**	13 (13%)	11 (24%)	2 (9%)	0 (0%)	9 (53%)^†^	<0.0001*	<0.0001^†^
**≥ moderate tricuspid regurgitation**	14 (14%)	8 (17%)	0 (0%)	1 (17%)	7 (41%)^†^	0.005*	0.001^†^
**Pulmonary artery systolic pressure, mm Hg**	38±11	47±19	36±10	47±26	61±10	0.001*	0.01^†^
**Right ventricular dysfunction**	9 (9%)	16 (35%)	0 (0%)	1 (17%)	10 (59%)^†^	<0.0001*	<0.0001^†^

Categorical data presented n (%), continuous data as mean±SD. NT‐proBNP indicates N‐terminal pro‐brain natriuretic peptide; NYHA, New York Heart Association; and TAVI, transcatheter aortic valve implantation.

* *P* value at first line testing.

^†^ *P* value after post hoc testing and Bonferroni correction for multiple comparisons.

Relevant periprocedural factors and 1‐year follow‐up echocardiographic data are summarized in Table 3. There was no difference in TAVI access site or choice of device between treatment responders and nonresponders. Measurements of technical success (postprocedural mean aortic pressure gradient, aortic valve area, and frequency of nontrivial aortic regurgitation) were similar across all groups. Compared with treatment responders, 1 year mortality was higher in nonresponders, with no significant difference between the groups according to baseline NT‐proBNP category. TAVI nonresponders with high baseline NT‐proBNP (>10 000 ng/L) had higher indexed LV mass and worse LV systolic function at 1 year. Persistent ≥ grade II diastolic dysfunction was more common in TAVI nonresponders with high baseline NT‐proBNP, who also exhibited increased LV filling pressures at 1 year. Concomitant significant mitral regurgitation, tricuspid regurgitation, and right ventricular dysfunction were also all more common at 1 year in TAVI nonresponders with high baseline NT‐proBNP.

**Table 3:** Between Group Differences in Procedural and 1‐Year Follow‐Up Characteristics of TAVI Symptomatic Responders Versus Nonresponders

	Responders	Nonresponders (All)	Nonresponders (Grouped by NT‐proBNP category)			*P* Value	Adjusted *P* Value
			Low NT‐proBNP	Midrange NT‐proBNP	High NT‐proBNP		
	n=98	n=46	NT‐proBNP <800 ng/L (n=23, 50%)	800 < NT‐proBNP <10 000 (n=6, 13%)	NT‐proBNP >10 000 ng/L (n=17, 37%)		
Nontransfemoral access	13 (13%)	6 (13%)	4 (17%)	0 (0%)	2 (12%)	0.732	
Balloon expandable valve	88 (90%)	39 (85%)	19 (83%)	5 (83%)	15 (88%)	0.785	
Self‐expanding valve	10 (10%)	7 (15%)	4 (17%)	1 (17%)	2 (12%)	0.785	
1‐y mortality	7 (7%)	10 (22%)	5 (22%)	1 (17%)	4 (24%)	0.017*	0.002^†^
≥ mild paravalvular aortic regurgitation	27 (28%)	11 (24%)	6 (26%)	1 (17%)	4 (24%)	0.283	
Mean gradient, mm Hg	12±6	14±7	16±7	13±8	13±9	0.224	
Aortic valve area, cm^2^	1.8±0.3	1.7±0.5	1.6±0.4	1.5±0.5	1.6±0.6	0.707	
**Left ventricular ejection fraction**	56±9	53±10	58±4	55±9	46±13^†^	0.002*	0.003^†^
**Left ventricular mass index, g/m^2^**	101±32	112±34	94±21	125±49	125±32^†^	0.026*	0.049^†^
Nonregression of left ventricular mass	19 (19%)	14 (30%)	7 (30%)	2 (33%)	5 (29%)	0.848	
**Estimated left ventricular filling pressure**	12±5	15±6	15±5	19±6	23±7^†^	<0.0001*	<0.0001^†^
**≥ Grade II diastolic dysfunction**	18 (18%)	16 (35%)	5 (22%)	1 (33%)	10 (59%)^†^	<0.0001*	0.001^†^
**≥ moderate mitral regurgitation**	7 (7%)	10 (22%)	2 (9%)	1 (17%)	7 (41%)^†^	0.001*	<0.0001^†^
**≥ moderate tricuspid regurgitation**	9 (9%)	10 (22%)	0 (0%)	1 (17%)	9 (53%)^†^	*	<0.0001^†^
**Pulmonary artery systolic pressure, mm Hg**	35±8	48±20	35±8	40±15	62±20^†^	<0.0001*	0.003^†^
**Right ventricular dysfunction**	12 (12%)	16 (35%)	4 (17%)	2 (33%)	10 (59%)^†^	<0.0001*	<0.0001^†^

Categorical data presented n (%), continuous data as mean±SD. NT‐proBNP indicates N‐terminal pro‐brain natriuretic peptide; and TAVI, transcatheter aortic valve implantation.

* *P* value at first line testing.

^†^ *P* value after post hoc testing and Bonferroni correction for multiple comparisons.

### Predictors of Symptomatic Response to TAVI

Factors associated with symptomatic response to TAVI are detailed in Table 4. In univariable analyses, baseline predictors of no improvement in NYHA functional class at 1 year were plasma NT‐proBNP >10 000 ng/L, NT‐proBNP <800 ng/L, concomitant chronic lung disease, at least moderate mitral regurgitation, pulmonary artery systolic pressure, and right ventricular dysfunction. After adjustment for confounders on multivariable analysis, baseline NT‐proBNP >10 000 and <800 ng/L remained the only factors demonstrating an independent association with nonresponse to TAVI at 1 year. This independent association between NT‐proBNP thresholds and functional outcome was preserved in an additional sensitivity analysis that considered discrete cutoffs of the continuous variables estimated glomerular filtration rate, left ventricular ejection fraction, pulmonary artery systolic pressure, and estimated left ventricular filling pressure (Table S1).

**Table 4:** Univariable and Multivariable Analyses of Baseline Factors Associated With Symptomatic Nonresponse to TAVI

	Univariable		Multivariable	
	OR (95% CI)	*P* Value	OR (95% CI)	*P* Value
NT‐proBNP >10 000 ng/L	113.33 (21.04–610.37)	<0.0001*	62.65 (6.47–664.99)	0.004^†^
NT‐proBNP <800 ng/L	29.1 (6.73–54.59)	<0.0001*	40.68 (7.36–738.21)	0.0001^†^
Estimated glomerular filtration rate (per 1 mL/min increase)	0.98 (0.97–1.01)	0.989	1.00 (0.96–1.04)	0.869
Chronic lung disease	2.78 (1.27–6.11)	0.010*	6.63 (0.83–53.34)	0.075
Atrial fibrillation	0.57 (0.24–1.35)	0.205	0.13 (0.01–3.09)	0.130
Left ventricular ejection fraction (per 1% increase)	0.98 (0.95–1.04)	0.298	0.99 (0.89–1.10)	0.991
Estimated left ventricular filling pressure	1.02 (0.97–1.16)	0.417	0.98 (0.97–1.10)	0.707
≥ Grade II diastolic dysfunction	1.79 (0.87–3.68)	0.109	1.18 (0.09–2.73)	0.138
≥ moderate mitral regurgitation	3.35 (1.18–9.44)	0.023*	4.30 (0.37–49.07)	0.240
≥ moderate tricuspid regurgitation	2.54 (0.96–6.76)	0.061	4.36 (1.06–11.80)	0.382
Pulmonary artery systolic pressure (per 1 mm Hg increase)	1.05 (1.02–1.10)	0.003*	1.05 (0.98–1.12)	0.205
Right ventricular dysfunction	3.86 (1.45–10.30)	0.007*	1.26 (0.15–10.86)	0.832

NT‐proBNP indicates N‐terminal pro‐brain natriuretic peptide; OR, odds ratio; and TAVI, transcatheter aortic valve implantation.

* indicates significant univariable association.

^†^ significant multivariable association.

## Discussion

The current report examines the relationship between baseline NT‐proBNP and TAVI symptomatic outcome at 1 year (∆NYHA functional class) from a novel perspective, demonstrating that both very high (>10 000 ng/L) and low (<800 ng/L) levels correlate strongly with poor functional improvement from the procedure (defined as ∆NYHA ≤0) in 144 patients undergoing TAVI at a single high‐volume center with comprehensive clinical and echocardiographic follow‐up. After adjustment for known confounders (including: renal function, left ventricular ejection fraction, concomitant valve disease, lung disease, atrial fibrillation, diastolic dysfunction, pulmonary hypertension, and right ventricular dysfunction), baseline NT‐proBNP levels outside this “sweet‐spot” range were the only factors independently associated with poor symptomatic response to TAVI. Very high (>10 000 ng/L; OR, 65; 95% CI, 6–664) and low (<800 ng/L; OR, 73; 95% CI, 7–738) NT‐proBNP levels both strongly predicted poor functional outcome with high diagnostic accuracy (sensitivity 88%, specificity 83%, positive predictive value 72%, negative predictive value 93%).

Prior retrospective studies examining the relationship between baseline natriuretic peptide levels and TAVI clinical outcomes have been inconsistent and yielded conflicting results. A number have described an association between baseline BNP/NT‐proBNP and postprocedural mortality,^14^, ^15^, ^27^, ^28^, ^29^, ^30^, ^31^ whereas others have not.^16^, ^17^, ^18^, ^19^, ^20^, ^32^ Crucially, all assumed a linear relationship. It is relevant, therefore, that a more recent large‐scale analysis of the PARTNER‐2 (Placement of Aortic Transcatheter Valves) trial cohort described a bimodal distribution of clinical events against baseline BNP and highlighted increased cardiovascular mortality in patients with intermediate surgical risk and preserved LV systolic function with both low (<50 ng/L) and high (≥400 ng/L) baseline levels.^33^

The present analysis builds on this latest work and challenges the previous assumption of a linear relationship between baseline natriuretic peptide levels and symptom response to TAVI. To the best of our knowledge, this association has not been previously described. The increased risk of persistent breathlessness in patients with a low baseline NT‐proBNP (<800 ng/L), despite effective relief of LV outflow obstruction via technically successful TAVI, may well suggest an alternative predominant cause for their symptoms. Indeed, this finding may be partially explained by the high prevalence of chronic lung disease in this group (52%)—a recognized risk factor for poor TAVI outcome and a frequent diagnostic dilemma for clinicians discerning the relative contributions of aortic stenosis and respiratory disease to an individual patient's presentation.^34^, ^35^ Identification of a marker threshold to predict futility of symptom relief with a high degree of specificity in this cohort is therefore of high clinical value.

In contrast, because natriuretic peptide levels correlate with the extent of LV hypertrophy,^36^, ^37^ and very low levels have been associated with myocardial injury,^38^, ^39^ it has been speculated that low circulating BNP/NT‐proBNP levels in patients with AS may represent an insufficiently developed compensatory mechanism for increased wall stress and vulnerability to irreversible myocardial fibrosis—a substrate for poor post‐TAVI outcomes.^33^ This concept is not supported by the diastolic filling parameters at 1‐year follow‐up in the present data set, where average estimated left ventricular filling pressure in treatment nonresponders with NT‐proBNP <800 ng/L (15±5) was similar to those patients with symptomatic improvement (12±5) but may merit attention in future work.

In the present study, patients with a very high baseline NT‐proBNP level (>10 000 ng/L) and poor symptom response to TAVI exhibited frequent concomitant cardiac damage (impaired LV systolic function, raised LV filling pressures, significant mitral and tricuspid regurgitation, elevated pulmonary artery systolic pressure, and impaired RV function), which persisted after successful TAVI. This finding is in agreement with recent analyses suggesting a strong relationship between the extent of preoperative secondary “extra‐aortic cardiac damage” and poor postprocedural outcomes following TAVI or surgical aortic valve replacement.^40^, ^41^, ^42^ Whether this structural damage is consequential (ie, progressive LV adverse remodeling and its downstream sequelae) or merely associated is not possible to discern from the present data. However, irrespective of the underlying etiology, a cardiac injury threshold beyond which relief of LV outflow tract obstruction fails to induce effective recovery is highly relevant to patient selection and risk prediction; particularly as TAVI continues to expand into younger, lower risk populations (with fewer noncardiac comorbidities), where the extent of baseline cardiac damage may arguably play an even greater role. Characterizing this threshold with a specific serum NT‐proBNP level (and appropriate diagnostic accuracy) is therefore highly useful and may also avoid expensive, futile procedures in patients who will derive no clinical benefit.

A wealth of evidence suggests that TAVI provides both quality and quantity of life benefits for the majority of patients with symptomatic AS. Yet a significant minority fail to realize these even after a technically successful procedure.^43^ The priorities of individual patients with severe symptomatic AS are likely to be variable. For those relatively unimpeded by symptoms at baseline, the priorities may well be reduction in mortality risk and maintenance of an acceptable quality of life. However, as previously demonstrated in elderly heart failure populations, patients greatly limited by symptoms are highly likely to place greatest value on the extent of symptomatic improvement.^44^, ^45^ At present, our ability to hold informed discussions with patients regarding the anticipated symptomatic outcomes of TAVI is impeded by the modest predictive capacity of currently available TAVI‐specific risk stratification tools and their failure to address symptom improvement or change in functional capacity. The present analysis identifies a clear link between TAVI futility and thresholds of a readily available marker (NT‐proBNP), which may be easily incorporated into future risk stratification tools. Further study will ascertain whether this may be useful in informing patient selection.

### Limitations

Our findings in this relatively small, single‐center study should be considered hypothesis‐generating and interpreted with attendant caution. Further examination of the findings in an independent external cohort is warranted. Frailty status (an important factor determining TAVI outcomes) was not routinely recorded in the present data set and would certainly merit assessment in future work. Finally, although a well‐established tool within cardiology, the limitations of the subjective, categorical NYHA functional classification system are well documented. Quality of life metrics and other functional data (eg, 6‐minute walk test distance) may be more sensitive metrics of functional change than NYHA class and systematic collection of these variables would be desirable in a future data set.

## Conclusions

Baseline NT‐proBNP is a useful prognostic marker in predicting the absence of symptom relief following TAVI. Demonstrating an inverted‐U relationship with improved symptom class at 1 year, patients with baseline NT‐proBNP levels <800 or >10 000 ng/L derived no functional improvement post‐procedure, suggesting an alternative cause for symptoms in the former scenario and an irrevocably diseased left ventricle in the latter. Further prospective evaluation of this relationship is warranted allied with more sensitive objective metrics of functional status.

## Sources of Funding

None.

## Disclosures

Drs Allen, Joseph, and McConkey are supported by a British Heart Foundation Clinical Research Training Fellowships (FS/18/48/33745, FS/15/52/31587, FS/16/51/32365, respectively). Dr Patterson is supported by a National Institute for Health Research Academic Clinical Lectureship. Professor Redwood has received speaker fees from Edwards Lifesciences and has served as an international advisory board member for Medtronic. Professor Prendergast has received speaker fees from Edwards Lifesciences. The remaining authors have no disclosures to report.

## Supporting information


**Table S1**
**Figure S1**
