# Supplementary material for: Baseline NT‐proBNP Accurately Predicts Symptom Response to Transcatheter Aortic Valve Implantation
Source: J Am Heart Assoc. 2020 Nov 26;9(23):e017574. doi: 10.1161/JAHA.120.017574 (PMC7763793; doi:10.1161/JAHA.120.017574)

Supplemental Material

Table S1. Univariable and multivariable analyses of baseline factors associated with symptomatic non-response to TAVI.

	Univariable		Multivariable	
	OR (95% CI)	p-value	OR (95% CI)	p-value
NT-proBNP > 10,000 ng/L	**113.33 (21.04 – 610.37)**	**<0.0001***	**36.21 (17.51 – 186.04)**	**<0.0001****
NT-proBNP < 800 ng/L	**29.1 (6.73 – 54.59)**	**<0.0001***	**16.11 (6.62 – 103.00)**	**<0.0001****
eGFR < 60 ml/min	1.35 (0.67 – 2.75)	0.404	0.76 (0.23 – 2.47)	0.869
Chronic lung disease	**2.78 (1.27 – 6.11)**	**0.010***	1.85 (0.49 – 6.97)	0.130
Atrial fibrillation	0.57 (0.24 – 1.35)	0.205	1.97 (0.32 – 12.1)	0.461
Left ventricular ejection fraction < 50%	1.00 (0.43 – 2.35)	0.500	1.21 (0.22 – 6.65)	0.826
E/e' > 14	1.18 (0.59 – 2.37)	0.637	1.71 (0.16 – 3.11)	0.707
≥ Grade II diastolic dysfunction	1.79 (0.87 – 3.68)	0.109	0.70 (0.15 – 3.30)	0.659
≥ moderate MR	**3.35 (1.18 – 9.44)**	**0.023***	0.60 (0.10 – 3.57)	0.578
≥ moderate TR	2.54 (0.96 – 6.76)	0.061	13.34 (0.58 – 30.51)	0.105
Pulmonary artery systolic pressure > 60 mmHg	**3.92 (1.09 – 14.16)**	**0.037***	0.96 (0.02 – 5.23)	0.251
RV dysfunction	**3.86 (1.45 – 10.30)**	**0.007***	1.71 (0.30 – 12.51)	0.082

eGFR – estimated glomerular filtration rate; MR – mitral regurgitation; TR – tricuspid regurgitation; RV – right ventricular.

Figure S1. Symptom status defined by New York Heart Association (NYHA) functional class at baseline and one year after TAVI in: A: Treatment responders and non-responders (defined as ΔNYHA≤0). B: Treatment responders and non-responders by baseline NT-proBNP category.

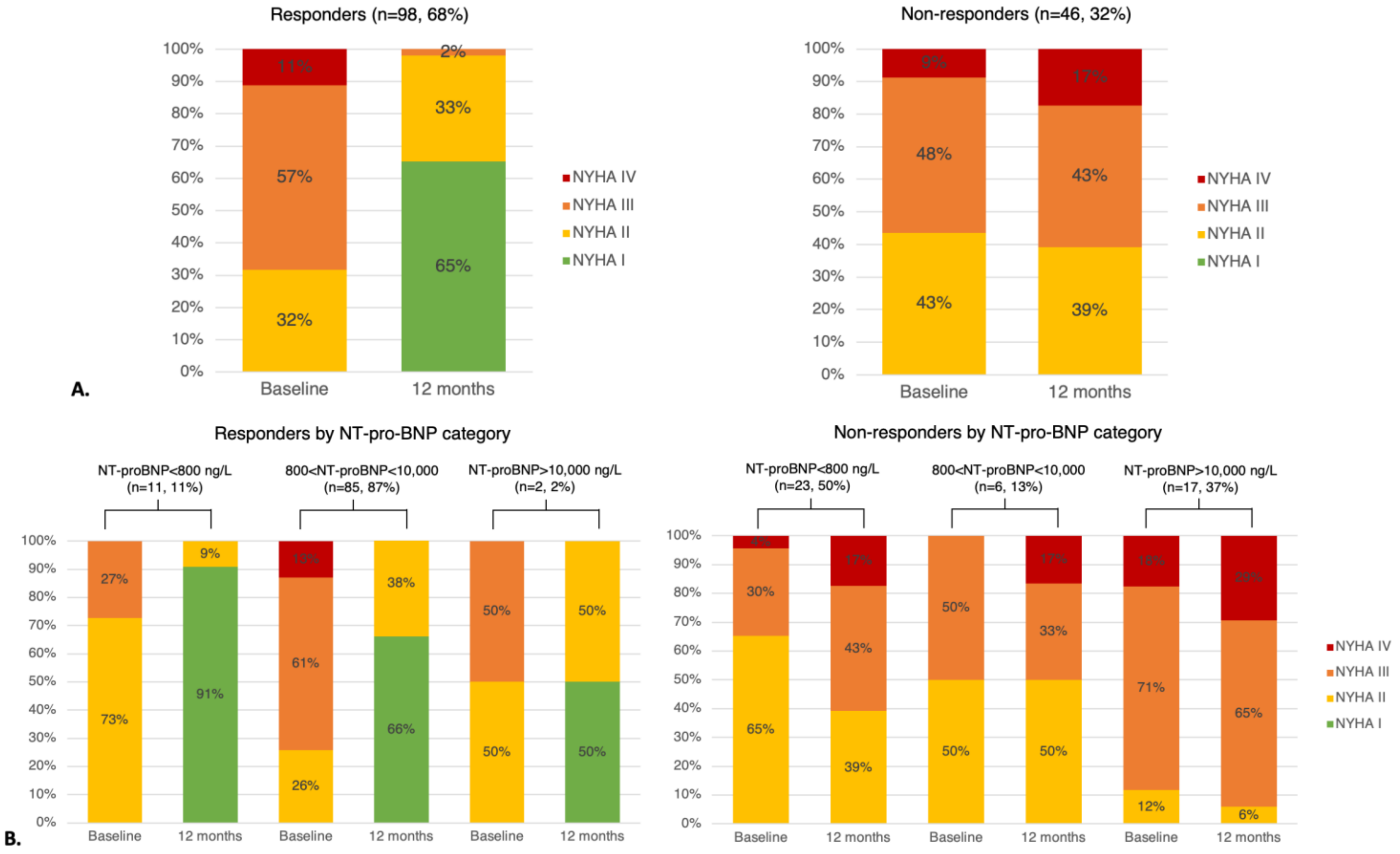

Supplement: Supplementary file 1 — Table S1 Figure S1 [file JAH3-9-e017574-s001.pdf]
